# Interleukin-1 as an Injury Signal Mobilizes Retinyl Esters in Hepatic Stellate Cells through Down Regulation of Lecithin Retinol Acyltransferase

**DOI:** 10.1371/journal.pone.0026644

**Published:** 2011-11-04

**Authors:** Yujiro Kida, Zanxian Xia, Sujun Zheng, Nicholas M. Mordwinkin, Stan G. Louie, Song Guo Zheng, Min Feng, Hongbo Shi, Zhongping Duan, Yuan-Ping Han

**Affiliations:** 1 Department of Surgery, Keck School of Medicine of the University of Southern California, Los Angeles, California, United States of America; 2 Beijing YouAn Hospital, Capital Medical University, Beijing, The People's Republic of China; 3 School of Pharmacy, University of Southern California, Los Angeles, California, United States of America; 4 Department of Medicine, Keck School of Medicine of the University of Southern California, Los Angeles, California, United States of America; 5 Beijing Hepo Medical Research Institute, Beijing, The People's Republic of China; 6 Department of Pathology, Keck School of Medicine of the University of Southern California, Los Angeles, California, United States of America; Vanderbilt University Medical Center, United States of America

## Abstract

Retinoids are mostly stored as retinyl esters in hepatic stellate cells (HSCs) through esterification of retinol and fatty acid, catalyzed by lecithin-retinol acyltransferase (LRAT). This study is designated to address how retinyl esters are mobilized in liver injury for tissue repair and wound healing. Initially, we speculated that acute inflammatory cytokines may act as injury signal to mobilize retinyl esters by down-regulation of LRAT in HSCs. By examining a panel of cytokines we found interleukin-1 (IL-1) can potently down-regulate mRNA and protein levels of LRAT, resulting in mobilization of retinyl esters in primary rat HSCs. To simulate the microenvironment in the space of Disse, HSCs were embedded in three-dimensional extracellular matrix, by which HSCs retaine quiescent phenotypes, indicated by up-regulation of LRAT and accumulation of lipid droplets. Upon IL-1 stimulation, LRAT expression went down together with mobilization of lipid droplets. Secreted factors from Kupffer cells were able to suppress LRAT expression in HSCs, which was neutralized by IL-1 receptor antagonist. To explore the underlying mechanism we noted that the stability of LRAT protein is not significantly regulated by IL-1, indicating the regulation is likely at transcriptional level. Indeed, we found that IL-1 failed to down-regulate recombinant LRAT protein expressed in HSCs by adenovirus, while transcription of endogenous LRAT was promptly decreased. Following liver damage, IL-1 was promptly elevated in a close pace with down-regulation of LRAT transcription, implying their causative relationship. After administration of IL-1, retinyl ester levels in the liver, as measured by LC/MS/MS, decreased in association with down-regulation of LRAT. Likewise, IL-1 receptor knockout mice were protected from injury-induced down-regulation of LRAT. In summary, we identified IL-1 as an injury signal to mobilize retinyl ester in HSCs through down-regulation of LRAT, implying a mechanism governing transition from hepatic injury to wound healing.

## Introduction

Hepatic stellate cells (HSCs) are vitamin A (retinol)-storing pericytes, residing in the space of Disse between sinusoidal endothelial cells and hepatocytes [1]. Lipid droplets in quiescent HSCs consist of 30 to 40% retinyl ester and other components such as triglyceride, cholesterol, phospholipids, and free fatty acids [2]. In the liver, hepatocytes absorb retinoids as retinyl ester, which is comprised of chylomicron remnant and combined with retinol binding protein (RBP) [3] [4]. In the cytoplasm of hepatocytes, retinyl ester is hydrolyzed to retinol and transferred from RBP to cellular retinol binding protein-I (CRBP-I) [5]. Binding with CRBP-I in HSCs, the retinol is esterified with fatty acids by two enzymes, namely lecithin retinol acyltransferase (LRAT) or acyl-coenzyme-A retinol acyltransferase (ARAT) using two different coenzyme factors [6]. The important role of LRAT in retinoid storage has been demonstrated by the gene knockout mice, showing striking absence of retinyl ester containing lipid droplets in HSCs [7]. Among the retinoid components, retinoic acid is the most active biological compound and is essential for embryonic development of all chordate animals. Once mobilized from retinyl ester, the free retinol can be sequentially oxidized to retinoic acid by retinol dehydrogenases (i.e. Rdh10) that oxidize retinol to retinaldehyde, and retinaldehyde dehydrogenases (Raldh1, Raldh2, and Raldh3) that metabolize retinaldehyde to retinoic acid [8]. The elevated retinoic acid may help wound closure by immune regulation/suppression, including induction of regulatory T cells from naive CD4^+^ cells as observed by one of the authors [9]. Moreover, retinoic acid can suppress pro-inflammatory cytokine-induced matrix metalloproteinases (MMPs) by down-regulating JNK-AP-1 signaling [10]. Administration of all-trans retinoic acid alleviated the liver injury and reduced an incidence of death following hepatic failure [11].

Upon liver injury, HSCs are promptly mobilized for wound healing regardless of any disease courses [12]. An early study showed PDGF in the conditioned medium from Kupffer cells contributed in part to mobilization of HSC stored retinoid. However, it is largely unknown as to whether and how injury signal mobilizes retinoid storage in HSCs during liver damage. Our previous study has revealed that interleukin-1 (IL-1) plays important roles in orchestrating liver injury and wound healing through production of MMPs by HSCs [13]. Recently, we showed IL-1 coordinates the progression from hepatic injury to wound healing and early fibrosis through HSC activation [14]. Thus, we reasoned that the same injury signal, IL-1, might also mobilize stored retinyl ester in HSCs in acute phase of liver injury. Released retinol and its derived products from HSCs may contribute to wound healing and tissue repair.

## Results

### IL-1 down regulates LRAT in rat HSCs

**Figure 1:**
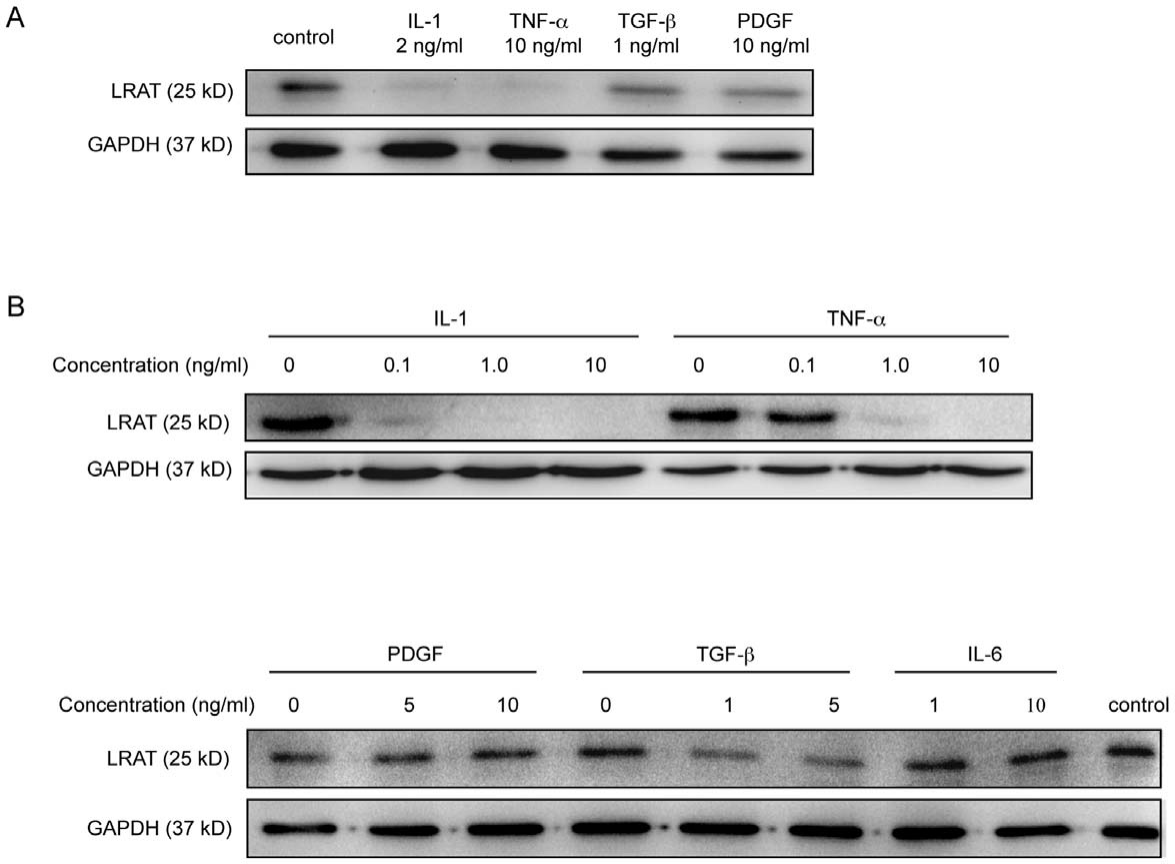
Interleukin-1 potently suppresses LRAT protein in primary rat hepatic stellate cells. Hepatic stellate cells (HSCs) were isolated from rat liver. The cells were recovered for 2 days in DMEM with 10% FBS, during which the cells maintain quiescent features as indicated by lipid droplets and stellar shape. The day-2 cells were seeded in 24-well plate (4×10^5^/well) for another day, followed by treatment with cytokines or growth factors for additional 24 hours. The protein levels of LRAT in HSCs were analyzed by Western blot. (A) The effects of various cytokines and growth factors on LRAT protein expression were measured in comparison with GAPDH. (B) The dose effects of IL-1α and TNF –α on LRAT protein expression were compared (upper panel). The dose effects of PDGF, TGF –β and IL-6 on LRAT protein expression were compared (lower panel). GAPDH was used as loading control.

**Figure 2:**
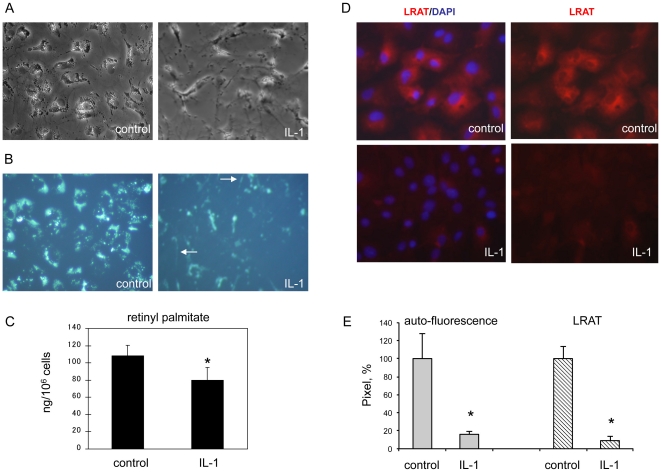
Interleukin-1 induced mobilization of retinyl ester in primary rat hepatic stellate cells is related to down-regulation of LRAT. Primary rat HSCs were cultured on plastic plate and treated with or without IL-1α (10 ng/ml) for 3 days. (A) Phase contrast images of HSCs. (B) Vitamin A auto-fluorescence in HSCs under UV excitation. Arrows indicate the small droplets that undergo mobilization along cytoplasmic processes as visualized by UV excitation. (C) Retinyl palmitate storage was measured by LC/MS/MS analysis, by which all-trans retinoic acid was used as an internal control for sample normalization. Results are expressed as ng/10^6^ cells. *P<0.05 (n = 6). (D) Immunofluorescence staining for LRAT (red) in HSCs. Nuclei are indicated by DAPI staining (blue). Original magnification ×200. (E) Densitometry (pixel) analysis of 5 independent images or fields. *P<0.05.

We reasoned that mobilization of retinyl ester storage for wound healing is mediated by injury signal from acute phase cytokines or growth factors. To test our hypothesis, primary rat HSCs were isolated from Wistar rats. Within a few days after isolation, HSCs retain retinoid droplets and maintain quiescent phenotypes as indicated by small nuclei and stellar shape with strong auto-fluorescence under ultraviolet (UV) excitation. Primacy HSCs were exposed to a panel of factors including IL-1α, IL-6, tumor necrosis factor (TNF)-α, transforming growth factor (TGF)-β1, and platelet derived growth factor (PDGF) for 24 hours. LRAT protein, as measured by Western blot analysis with a monoclonal antibody against the middle region of LRAT [15], was constitutively expressed in primary rat HSCs, in line with the presence of retinyl ester droplets viewed under UV excitation (Fig. 1A and Fig. 2B). Among tested factors, IL-1α and TNF-α were identified as the two most potent suppressors for LRAT protein expression. Then, we determined the efficacy of IL-1α and TNF-α on LRAT suppression. As shown, IL-1α at 0.1 ng/ml almost completely inhibited LRAT expression, while TNF-α required ten times higher concentration at 1 ng/ml to attain the same suppression (Fig. 1B). In additional study we measured the IC50 for the IL-1α-mediated suppression of LRAT at 0.05 ng/ml. TGF-β1, at high concentration, could partially down-regulate LRAT. PDGF and IL-6, on the other hand, were almost without any effect on LRAT down-regulation (Fig. 1B). In summary, we identified IL-1α as a potent suppressor to down-regulate LRAT expression in rat HSCs, implying a hierarchy role of IL-1 in mobilization of retinyl ester in liver injury for tissue repair and wound healing.

### IL-1 mobilizes vitamin A storage in rat HSCs

Next, we measured the effects of IL-1 on retinol storage in HSCs. Without cytokine exposure, isolated HSCs in the early days exhibited ample of lipid droplets as indicated by phase contrast microscopy (Fig. 2A). Under UV excitation, retinoids emit auto-fluorescence which is mostly enriched in peri-nuclear region. After exposure to IL-1α for 24 hours, big lipid droplets apparently started to dispense, and on day 3 most of big droplets were disappeared or mobilized (Fig. 2B). Consequently, fine smeared auto-fluorescent particles are aligned along cytoplasmic extensions by HSCs under IL-1 treatment (arrows in Fig. 2B). To accurately assess total storage of retinoids, we applied LC/MS/MS approach and quantitatively determined the amount of retinyl palmitate, the dominant form of stored retinoids in HSCs. After exposure to IL-1α for 3 days, the retinyl palmitate storage was significantly reduced in HSCs in comparison to the control treatment (Fig. 2C). Regarding the time course, LRAT was completely suppressed within 24 hours after IL-1α exposure, which occurs prior to retinyl ester depletion (Fig. 1A, 2B). In addition, we revealed cytoplasmic distribution of LRAT in control HSCs by immunofluorescence staining with a monoclonal antibody (Fig. 2D). LRAT protein and vitamin A droplets have a similar distributional pattern in HSCs (Fig. 2B, D). As expected, under IL-1α treatment, cellular expression of LRAT was markedly decreased, which is in agreement with decreased retinoid droplets and retinyl ester storage. The correlation between reduced auto-fluorescence of vitamin-A droplets and down-regulation of LRAT in HSCs was evident by pixel densitometry analysis of the images (Fig. 2E).

### IL-1 suppresses LRAT expression by HSCs embedded in three-dimensional extracellular matrix

**Figure 3:**
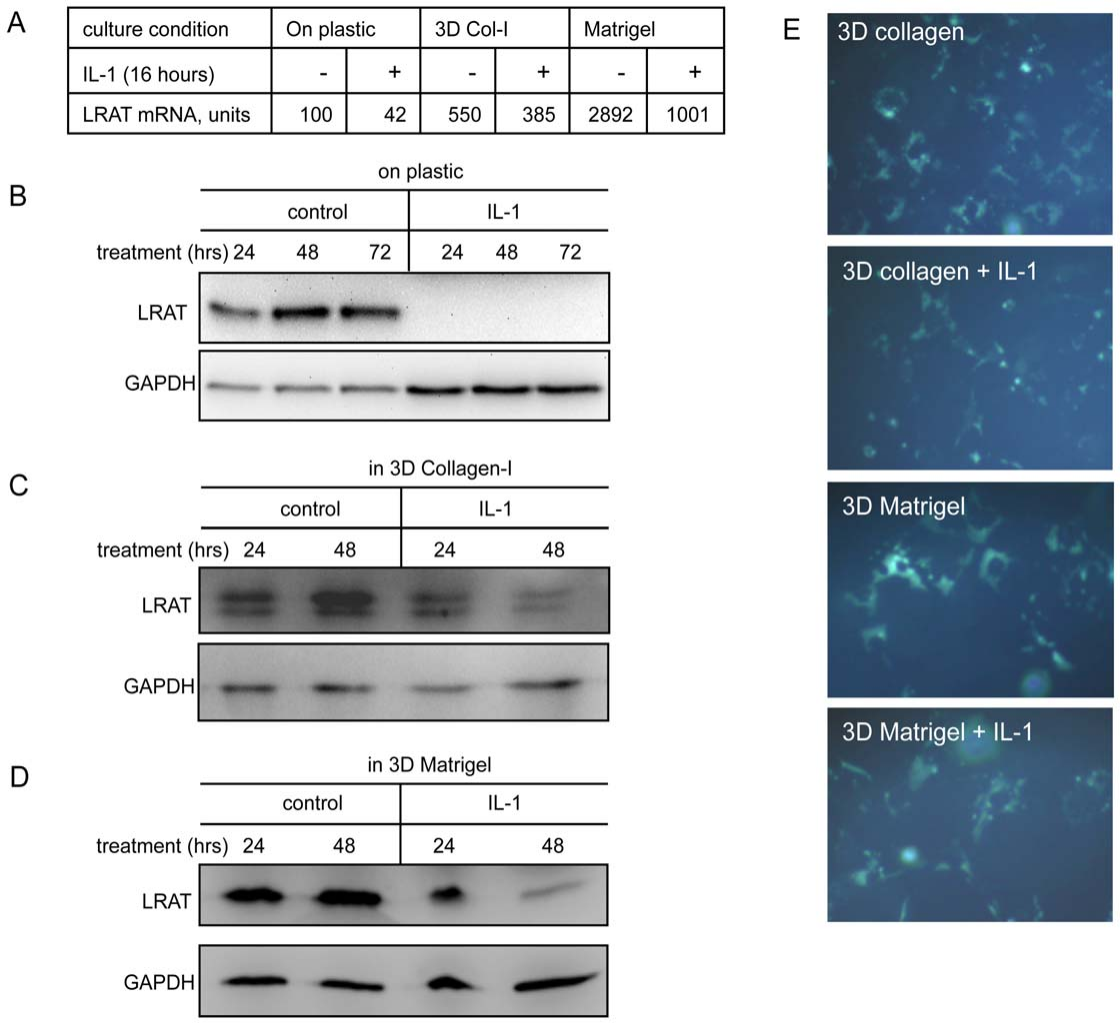
Interleukin-1 suppresses LRAT expression in primary rat hepatic stellate cells cultured in 3D ECM. (A) HSCs in equal number were seeded on plastic or embedded in 3D ECM (type I collagen or Matrigel, 1×10^5^ cells in 0.4 cm^3^). After recovery, the cultures were treated with or without IL-1 (10 ng/ml) for 18 hours. LRAT gene expression was measured by Affymetrix DNA microarray analysis, which has been extensively calibrated for many genes by qRT-PCR. (B–D) The levels of LRAT protein expressed by HSCs cultured on plastic or in 3D ECM. The effects of IL-1 treatments on LRAT expression were measured by Western blot analysis. GAPDH was used as loading control. (E) Vitamin A autofluorescence in the cultures was visualized by UV excitation for HSCs cultured in 3D ECM treated with or without IL-1 for 3 days. Original magnification ×200.

As the major extracellular matrix (ECM) producing cells in the liver, HSCs are lodged in ECM within the space of Disse [16]. We previously demonstrated that embedding of HSCs in three dimensional (3D) type-I collagen (Col-I) or 3D Matrigel recapitulates the sinusoidal microenvironment [13]. Further, sensing 3D ECM is critical for HSCs to make decision about whether and how much MMPs should be expressed in response to IL-1 stimulation in liver injury. Therefore, we examined an effect of ECM on LRAT expression and vitamin A storage as well as response of HSCs to IL-1. We prepared three types of culture conditions for primary rat HSCs as follows: (i) seeding HSCs on plastic, (ii) embedding HSCs in 3D Col-I matrix, (iii) embedding HSCs in 3D Matrigel. After overnight recovery, the cultures were exposed to IL-1α or not for additional 18 hours. As expected, mRNA of LRAT is at the lowest level by culture on plastic, and the highest level by culture in 3D Matrigel (Fig. 3A). Under IL-1α treatment, levels of LRAT mRNA went down in all of three culture conditions, showing the dominant role of IL-1 in down-regulation of LRAT. We then inspected protein levels of LRAT in HSCs by three culture conditions. When cultured on plastic, HSCs undergo “myofibroblastic trans-differentiation”, while LRAT protein was steadily increased, which may reflect a feedback compensation for the loss of retinoid storage. In 24 hours of treatment with IL-1, LRAT protein was totally vanished in HSCs cultured on plastic (Fig. 3B. To visualize the remained LRAT protein, we increased loading of IL-1 treated samples, where IL-1 still dominantly suppressed LRAT protein). Under IL-1 treatment, LRAT protein was significantly decreased by HSCs cultured in 3D Col-I or Matrigel, although residual LRAT protein was still detected after 48 hour treatment with IL-1α (Fig. 3C, 3D), which is in line with LRAT mRNA measurement (Fig. 3A). In 3D Matrigel, big lipid droplets showing auto-fluorescence were evident in HSCs. Under IL-1 stimulation, the droplets were apparently distributed or dispensed into smear microns in cytoplasmic extensions (Fig. 3E). Given the fact of prompt suppression of LRAT mRNA in 3D ECM by IL-1 but slow pace of down-regulation of LRAT protein, these results suggested that 3D ECM may modulate the turnover of LRAT expression.

### Down-regulation of LRAT by IL-1 at mRNA level

**Figure 4:**
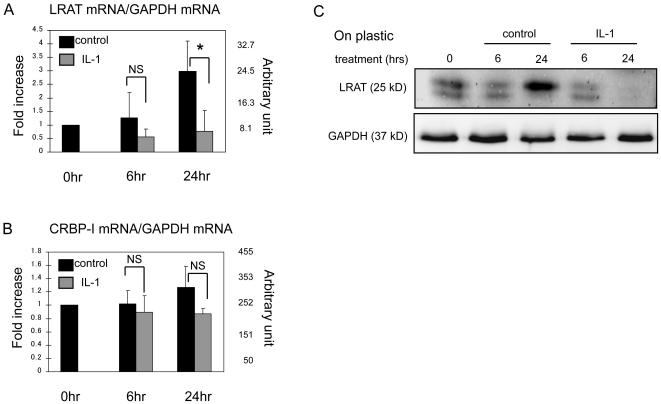
Interleukin-1 down-regulates LRAT mRNA in primary rat hepatic stellate cells. HSCs were cultured on plastic for indicated times with or without of IL-1 (10 ng/ml). The mRNA levels of LRAT (A) and CRBP-I (B) were measured by qRT-PCR. Results (mean ± SD) are expressed as relative folds of increase to 0 hr time point. NS: not significantly different, *P<0.05 (n = 3/group/time point) as significance. (C) The time course of LRAT protein in HSCs was assessed by Western blot. GAPDH: loading control.

Next we measured mRNA levels of LRAT and CRBP-I in response to IL-1 challenge in primary HSCs. Messenger RNA of LRAT was steadily elevated in the course of HSC culture, in agreement with increased protein level of LRAT (Fig. 3B, 4A). In contrast, mRNA of CRBP-I was constantly expressed. After 6 hours of IL-1 stimulation, mRNA of LRAT was apparently suppressed. At 24 hour time point, LRAT mRNA level was significantly decreased in IL-1-treated HSCs compared to the control, whereas CRBP-I mRNA level was not significantly affected by IL-1 (Fig. 4B).

### IL-1 does not regulate the stability of LRAT protein

**Figure 5:**
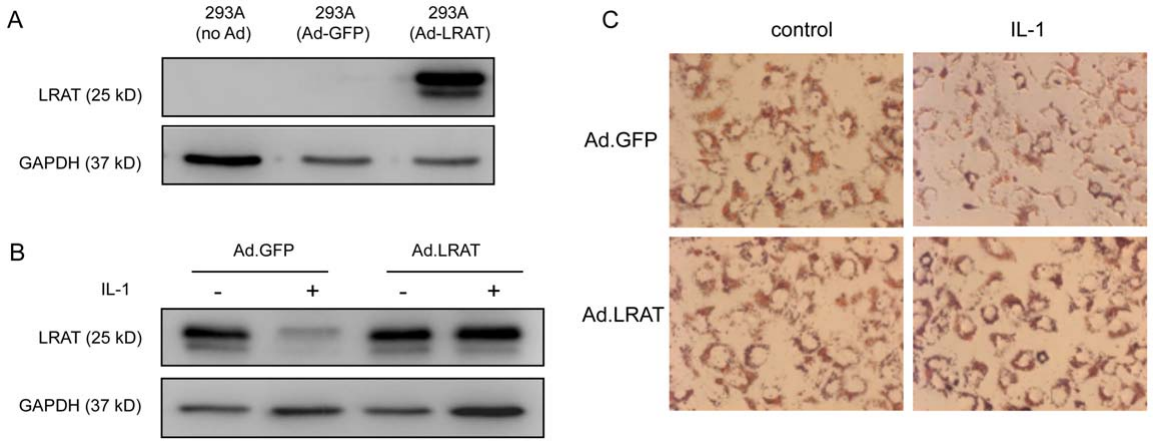
Interleukin-1 down regulates endogenous LRAT but not the ectopically expressed recombinant LRAT, indicating regulation is not at protein level. Coding region of rat *LRAT* gene was cloned and confirmed by DNA sequencing. Recombinant rat LRAT was expressed by adenoviral vector (Ad.LRAT) in 293A cells, and GFP expressing adenoviral vector (Ad.GFP) was used as the control. The expression of LRAT protein was assessed by Western blot analysis. GAPDH was used as loading control. (B) HSCs were transduced by adenoviral vectors with MOI at 100. Three days after transduction, HSCs were treated with or without IL-1 (10 ng/ml) for additional 3 days. As shown by Western blot analysis, the endogenous LART but not recombinant one is down-regulated by IL-1, indicating the regulation is not at protein level. (C) Oil red O staining for each group of HSCs. As shown, ectopic expression of recombinant LRAT attenuated IL-1-indcued mobilization of lipid droplets in HSCs. Original magnification ×200.

We compared the kinetics of LRAT mRNA with the kinetics of LRAT protein after IL-1 challenge. In the course of culture, both mRNA and protein levels of LRAT in HSCs were steadily increased in parallel (Fig. 4C). In IL-1 treated HSCs, down-regulation of LRAT mRNA closely mirrored its protein reduction (Fig. 4A, C), suggesting that the regulation is more likely at mRNA level rather than protein degradation. We also measured if IL-1 can enhance the degradation of LRAT protein. HSCs were treated by cycloheximide (20 µg/ml) together with or without IL-1α for a time course. In such experiment we did not find that IL-1 can significantly increase the degradation rate (data not shown), suggesting the key regulation by IL-1 is at transcriptional level. To further demonstrate the notion we cloned the full length of rat *LRAT* cDNA through reverse transcription of RNA extracts from rat HSCs, the open reading frame was subcloned into the adenovirus (Ad.LRAT). The size of expressed protein matched well the predicted size of LRAT protein, and can be recognized by a monoclonal antibody against a peptide derived from LRAT. Also in the 293A cells, the ectopically expressed LRAT exhibited doubled bands akin to endogenous LRAT (Fig. 5A). We then examined the ectopic LRAT and its regulation in HSCs. In a control condition, HSCs were transduced by adenoviral vector expressing GFP (Ad.GFP). Under IL-1α treatment, the endogenous LRAT was promptly decreased in control HSCs transduced with Ad.GFP (Fig. 5B). In contrast, IL-1α failed to down-regulate the ectopically expressed LRAT protein, which supports the notion that IL-1 does not promote degradation of LRAT protein. Retinoid droplets are composed of both retinyl ester and other lipids [3]. As revealed by Oil red O staining, the droplets were ample in the control HSCs, and were deprived under IL-1 exposure (Fig. 5C, right upper panel). HSCs, which ectopically express LRAT, were resistant to IL-1-induced mobilization of retinyl ester droplets, indicating LRAT as a key enzyme which is sufficient to restore retinoid storage (Fig. 5C, right lower panel).

### Secreted factor(s) from Kupffer cells suppressing LRAT in HSCs is identified as IL-1

**Figure 6:**
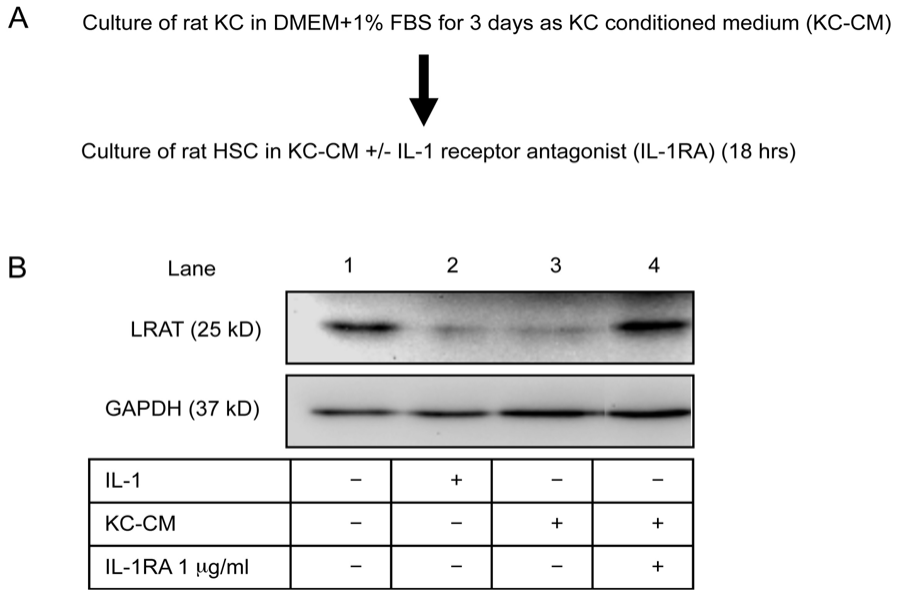
Identification of Kupffer cell secreted factor which suppresses LRAT expression in hepatic stellate cells as interleukin-1. (A) A diagram of experimental design. (B) Conditioned medium was prepared by culture of primary rat Kupffer cells (KCs) for 3 days. A portion of the conditioned medium was added to HSC culture for 18 hours. The inhibitory effects of KC secreted factors on LRAT expression by HSCs were analyzed by Western blot. To demonstrate the contribution of IL-1 in the conditioned medium of KCs, IL-1 receptor antagonist (IL-1RA, at 1 µg/ml) was added to the KC-CM prior to the treatment for HSCs. GAPDH: loading control.

Kupffer cells (KCs), resident liver macrophages, are the major source of inflammatory cytokines and injury signals in innate immunity. We speculated that KCs may transmit injury signals to activate HSCs for mobilization of retinoid in liver wound healing. Therefore, we inspected the effects of KCs, particularly their secreted factors, on the expression of LRAT in HSCs. Conditioned medium of primary rat KCs (KC-CM) was prepared and applied to HSCs for additional 18 hours (Fig. 6A). The expression of LRAT in HSCs was thoroughly suppressed by KC secreted factors presented in the conditioned medium. Application of IL-1 receptor antagonist, IL-1RA (1 µg/ml) to the conditioned medium fully neutralized the inhibitory function of KC-CM and restored the lost LRAT expression (Fig. 6B, lane 4). These results demonstrate IL-1 as a major injury signal secreted from KCs to suppress LRAT expression in HSCs. In another experiment we activated KCs by LPS (5 µg/ml) treatment, and the consequent conditioned medium had enormous inhibitory function to suppress LRAT in HSCs, which is in line with IL-1 expression (data not shown).

### IL-1 is a key mediator to down-regulate LRAT in liver injury

**Figure 7:**
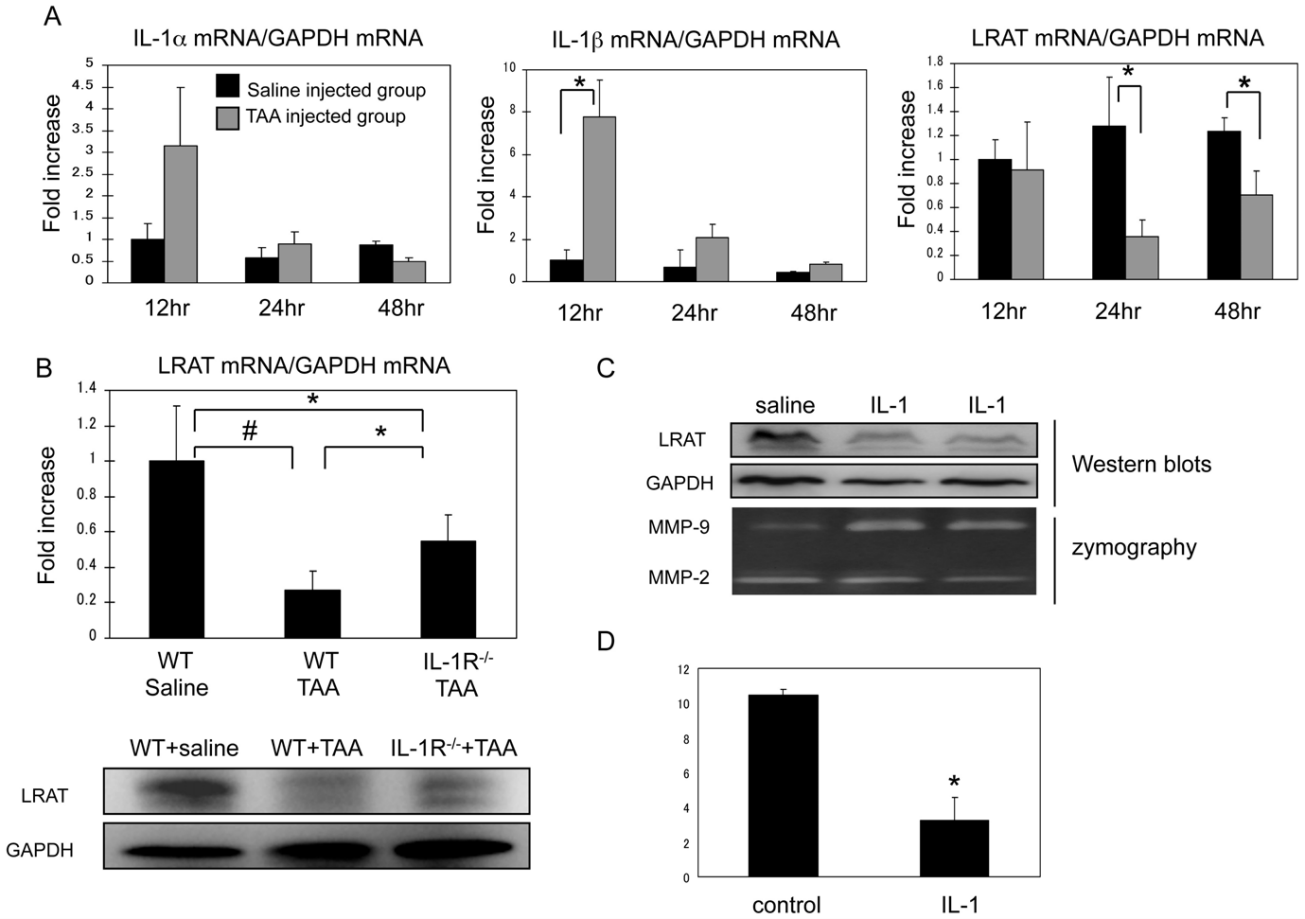
Interleukin-1 mediates down-regulation of LRAT in acute liver injury by mouse model. (A) Acute liver injury in WT mice was induced by challenge with TAA (0.2 mg/g body weight) via i.p. injection. Saline was used for control. Time course of mRNA levels for LRAT, IL-1α, and IL-1β in the liver was measured by qRT-PCR. Results (mean ± SD) are expressed as folds of increase related to the saline injected group (n = 5). (B) To determine the contribution of IL-1 toward injury-induced down-regulation of LRAT, IL-1R^−/−^ and WT mice were subjected to TAA injection. After 24 hours, mRNA level of LRAT was measured by qRT-PCR. Results (mean ± SD) are expressed as folds of increase relative to normal saline-injected WT mice. *P<0.05, #*P*<0.01. LRAT protein expression was assessed by Western blot. GAPDH: loading control. (C) WT mice were intravenously injected with recombinant IL-1α protein (20 ng) or saline for 24 hours. LRAT and MMP-9 levels in the liver tissues were examined by Western blot and zymography analysis respectively. (D) Hepatic retinyl palmitate levels after IL-1 injection for 24 hours were measured by LC/MS/MS using liver tissues from mice receiving intravenous injection of IL-1α (20 ng) or saline. *P<0.01 (n = 4/group).

We clearly demonstrated the multiple roles of IL-1 in down-regulation of LRAT mRNA, LRAT protein, and mobilization of retinoid storage in primary rat HSCs. Next, we investigated those events in animal models. Initially, we tested injury induced-regulation of LRAT. Wild type (WT) mice were given a single intraperitoneal injection of thioacetamide (TAA) at 0.2 mg/g body weight. We then measured mRNA levels of IL-1α, IL-1β and LRAT in liver samples harvested at 12, 24, and 48 hours after TAA injection. At 12 hour time point, the IL-1α and IL-1β mRNA levels were promptly increased to 3 and 8 folds respectively, whereas LRAT mRNA level was not yet decreased significantly at this time point (Fig. 7A). At 24 hour time point, LRAT mRNA level significantly dropped by 70% in comparison to the control. At 48 hours after injury, IL-1 expression returned to basal level, while LRAT mRNA conversely started restoration. The timely closed association between elevated IL-1 and down- regulated LRAT in liver injury indicates a possible causal relationship. To test this relationship, we performed two additional experiments. First, we examined hepatic LRAT expression at both mRNA and protein levels by IL-1 receptor^−/−^ (IL-1R^−/−^) mice and WT mice after liver injury. As expected, LRAT levels went down in WT mice after TAA injection, while IL-1R^−/−^ mice were partially, but significantly protected from TAA-induced LRAT down-regulation (Fig. 7B). Second, we explored if injection of IL-1 protein can sufficiently suppress LRAT expression in the liver in a way bypassing the injury. After 24 hours of intravenous injection with IL-1α (20 ng), LRAT protein in liver was substantially decreased (Fig.7 C). As a positive indicator of IL-1α effect on liver injury, we detected increased MMP-9 level in the liver tissues of IL-1α injected mice (Fig. 7C). Then, we measured the amount of retinyl palmitate in liver tissues of the mice subjected to IL-1α injection. As measured by LC/MS/MS, retinyl palmitate was significantly reduced in liver tissues from IL-1α injected mice in comparison to the control ones (Fig. 7D).

### Regulation of LRAT in HSCs from injured rat liver

**Figure 8:**
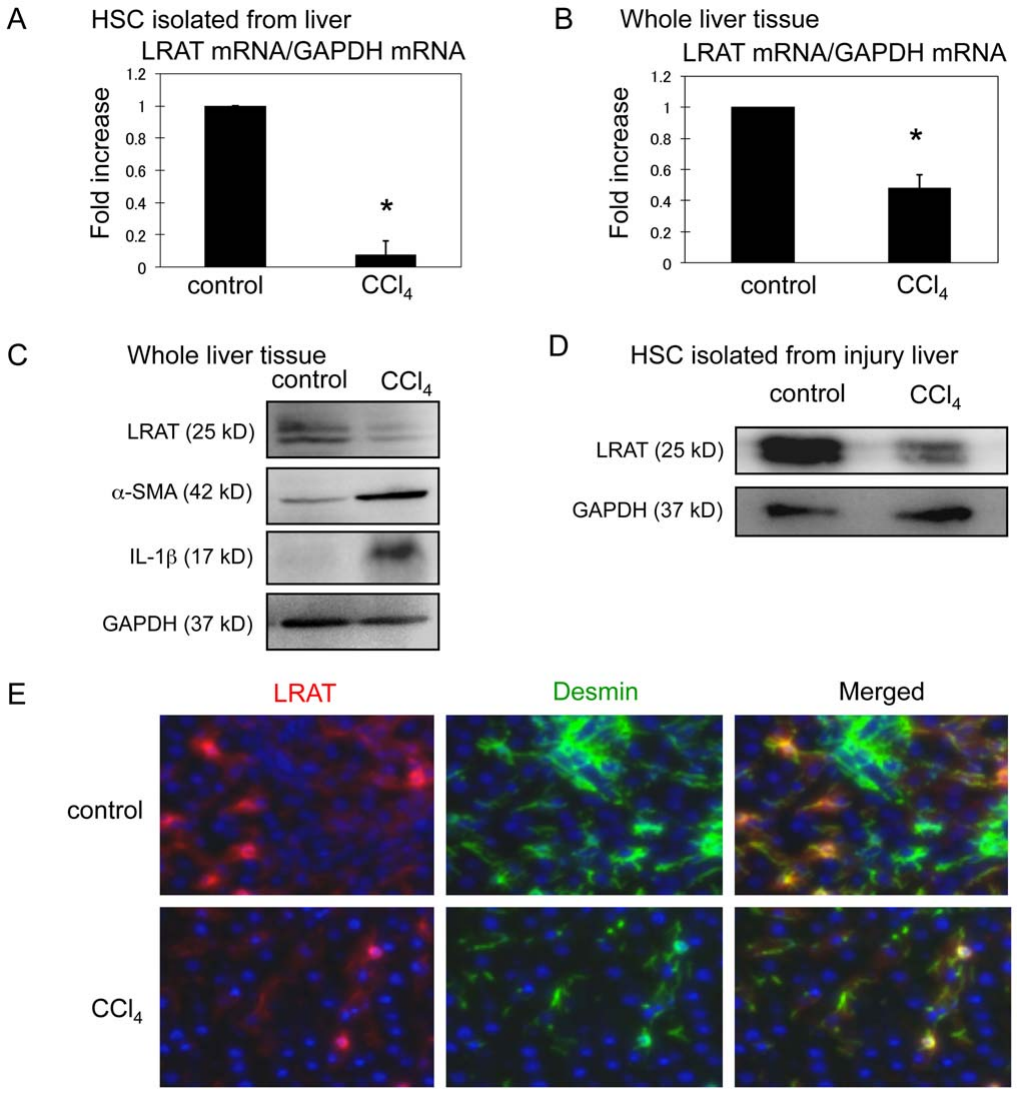
Down-regulation of LRAT in HSCs isolated from rat hepatic injury model. (A). Rats received a single subcutaneous injection of mineral oil (control) or CCl_4_. HSCs were isolated 24 hours after treatment. HSCs were cultured for additional 24 hours after isolation. LRAT mRNA levels in HSCs (A) and whole liver tissues (B) were measured by qRT-PCR. Results are expressed as folds of increase relative to control. *P<0.05 (n = 3). (C) Protein levels of LRAT, α-SMA, and IL-1β in whole liver tissue were analyzed by Western blot. GAPDH: loading control. (D) LRAT protein in HSCs isolated from control and injured liver was detected by Western blot. (E) Double immunofluorescence staining for LRAT (red) and desmin (green) in the liver of control and CCl_4_-injected rat. Original magnification ×200.

Primary HSCs used in our work were isolated from rats. Rat HSCs are more sensitive to IL-1 stimulation than HSCs from mice (Han et al., unpublished). We thus examined the regulation of LRAT in HSCs isolated from rat liver samples after a single injection of carbon tetrachloride (CCl_4_) or mineral oil as control. LRAT mRNA and its protein expression were substantially down-regulated in HSCs directly isolated from CCl_4_-injected rats than HSCs from the control (Fig. 8A). Similarly, in the whole liver, LRAT mRNA level was also suppressed by CCl_4_ injection (Fig. 8B). We also measured the association between reduction of LRAT and increase of IL-1 at protein levels. Activation of HSCs in liver injury was monitored by increased α-smooth muscle actin (α-SMA) protein. As expected, after a single injection of CCl_4_, IL-1β and α-SMA expressions were increased, whereas LRAT expression was decreased (Fig. 8C and D). Finally, we performed immunofluorescence staining for LRAT and desmin in rat livers. Desmin-positive HSCs also expressed LRAT in the liver of control rat, demonstrating the identity of HSCs by the two major markers (Fig. 8E). As expected, LRAT expression in desmin-positive HSCs was reduced by CCl_4_ induced-liver injury. Taken together, these results support our notion that IL-1 functions as a common injury signal to down-regulate LRAT expression in acute liver injury of both mice and rats.

## Discussion

HSCs (also called vitamin A-storing cells, lipocytes, hepatic pericytes, interstitial cells, fat-storing cells, and Ito cells) lodge in the space between hepatocytes and sinusoidal endothelial cells. In the normal liver, HSCs are assumed to have two major physiological functions. First, HSCs store 80% of whole body's retinoid in the form of retinyl ester in the cytoplasm [17]. Second, HSCs produce basement-membrane-like ECM scaffolds for mechanical support of the sinuosids. Upon injury, HSCs organize tissue repair and wound healing in multiple directions. For instance, when stimulated by inflammatory cytokines and in sensing of surrounding ECM, HSCs express a panel of MMPs, which can subsequently release cryptic growth factors stored in ECM. On the other hand, also under injury signals, HSCs mobilize stored retinyl esters and convert retinyl esters into retinol, which can be subsequently oxidized to retinoic acid to control and prevent over inflammation. Compelling evidence has demonstrated critical roles of retinoid acid in induction of peripheral regulatory T cells from naive T cells, which may subsequently quench inflammation [18]–[19]. A recent study showed that although having weak antigen presentation function, HSCs can prevent induced regulatory T cells from their conversion to Th17 cells [20]. Thus, presumably mobilization of retinoid in HSCs induced by IL-1 may contribute to induction of regulatory T cells for wound healing.

Vitamin A containing lipid droplets are regarded as a typical marker of quiescent HSCs in normal liver. It is well believed that mobilization or loss of retinoid storage in HSCs is one of key features for HSCs in wound healing [21]. Study showed that absence of lipid droplets in HSCs of LRAT^−/−^ mice does not enhance hepatic fibrosis, but decreases hepatic carcinogenesis [22]. Like triglyceride, vitamin A storage in HSCs is mediated by PPAR signaling [23]. However, it is still obscure that how vitamin A storage is mobilized by HSCs in acute phase of liver damage. To address this issue, we focused on LRAT, a well documented enzyme controlling esterification of retinol for its storage in HSCs. In 3D ECM, HSCs maintain quiescent state by up-regulation of LRAT accompanying accumulated retinoid droplets. One future direction is to address how 3D ECM up regulates LRAT expression level.

Present study showed, for the first time to our knowledge, that IL-1 can directly down-regulates LRAT expression, and mobilizes retinoid in primary rat HSCs. We reached this conclusion through a line of experimental approaches. First, we speculated that the acute cytokines in the early phase of tissue injury may serve as injury signals to mobilize retinyl ester. Through testing a panel of acute inflammatory cytokines and growth factors we identified IL-1 as a potent cytokine that can promptly mobilize and dispense retinoid droplets by the quiescent rat HSCs. Second, we demonstrated that IL-1 can promptly down regulate LRAT, which provides a molecular basis to explain how mobilization of retinoid occurs in HSCs and in acute liver injury. Third, to elaborate such notion we additionally found that KCs and their secreted mediators in the conditioned medium can elicit HSCs, resulting in LRAT down-regulation, suggesting patho-physiological cellular interaction in the liver wound healing. KCs are the major innate immune cells in the liver. Different from other macrophages, KCs in the liver are keen on immune tolerance [24]. After injury or infection, KCs can be promptly activated to secrete pro-inflammatory cytokines and other growth factors. Importantly, we demonstrated that IL-1 as a major, if not the exclusive signal from KCs to elicit HSCs for down regulation of LRAT. Such notion is based on results from *in vitro* neutralization by IL-1RA and *in vivo* depletion of KCs by liposomal clodronate (Feng et al., unpublished data). Fourth, the role of IL-1 in injury-induced mobilization of retinyl ester and down-regulation of LRAT was demonstrated here by variety of animal models. Our animal work started from establishing an inverse association between upraising of IL-1 and down-regulation of LRAT as well as mobilization of retinyl ester in the acute phase. Then, through gain-of-function analysis by direct administration of IL-1, we found IL-1 alone is sufficient to exert down-regulation of LRAT in the liver, in a way bypassing injury and inflammation. In a complementary approach through loss-of-function analysis, we showed that IL-1R^−/−^ mice are partially devoid of injury-induced down-regulation of LRAT. Finally, in a rat model challenged by different hepatic toxin we confirmed the relationship between IL-1 elevation and down-regulation of LRAT in the acute phase of liver injury. Taken together, these results prompted us to reach the conclusion that IL-1 may function as injury signal controlling mobilization of retiny ester through down-regulation of LRAT in liver injury. In addition to IL-1, other cytokines such as TNF-α, at a less extent, may also suppress LRAT expression *in vivo*. In fact, the partial protection of LRAT down-regulation in IL-1R deficient mice after injury also indicates contribution of other factors such as TNF-α. Since both IL-1 and TNF-α can be generated either from hepatic source or systemic way by other organs under inflammation, HSC derived retinol or retinoic acid may contribute to systemic wound healing for other organs. In particular, retinoic acid produced in acute phase of injury may participate in immune regulation/suppression through induction of regulatory T cells for termination of inflammation and wound closure.

Beside LRAT, there are several other critical factors controlling retinoids metabolism in HSCs. CRBP-I deficient mice showed an approximately 50% reduction of retinoid accumulation [25]. Such phenomena probably reflect an impaired delivery of retinol as a substrate for LRAT. Diacylglycerol acyltransferase-1 (DGAT-1) was reported to be highly expressed in HSCs [26] and function as an ARAT by using acetyl co-A as a carrier [27]. However, there was no difference in hepatic retinyl ester levels between DGAT-1 null mice and matched WT mice. Additionally, the role of LRAT as the major retinol esterification enzyme in liver was proved by LRAT^−/−^ mice [15]. Paradoxically, as Hellemans and coworkers reported [23], we observed time-dependent up-regulation of LRAT expression in culture activated HSCs, which may imply a compensatory feedback to restore the lost retinyl ester. Since HSCs mostly store retinoids transferrd from hepatocytes, HSCs can hardly uptake retinol without hepatocytes in our culture system. The up-regulation of LRAT expression does not necessarily reflect the quiescence of HSCs. *Vice versa*, in LRAT^−/−^ mice, which showed intense loss of hepatic retinoid storage, neither myofibroblastic change of HSC nor liver fibrogenesis was observed [7].

In Western blot for LRAT, we frequently observed doubled bands of LRAT. The predicted size of LRAT protein is 25 kDa. Although Moise and coworkers speculated that the lower band is most likely proteolytic products [28], nobody confirmed their speculation. When overexpressed in 293A cells, LRAT protein also showed the doublets (Fig. 5A). Therefore, the double bands are likely derived from coding region of LRAT cDNA, rather than RNA splicing. We still do not know the significance of the doublet and how they are generated. LRAT gene was constitutively transcribed by HSCs in basal state of the normal liver. Our data showed that ECM in 3D context can elevate LRAT expression at basal state, which is in line with increased storage of retiny ester, and coincides with quiescent phenotypes of HSCs in the normal liver. Thus it is possible that ECM produced by HSCs in autocrine manner may enforce the cells to express LRAT and accumulate retinyl ester. This is particularly reasonable in terms of microenvironment that determines gene expression through a negative feedback loop. However, up-regulation of LRAT, as observed by HSC activation in current study, is not sufficient to promote retinol esterification. Other factors such as substrate availability and carrier proteins are also required for storage of retinyl esters. Although PPARβ is reported to be an inducer of LRAT expression in HSCs [23], regulators of LRAT expression are largely unknown at present.

In present study, it is still unknown how IL-1 suppresses LRAT expression at molecular level. Under IL-1 stimulation, both mRNA and protein of LRAT decayed by an intimate pace. These results suggest that IL-1 regulates LRAT expression mainly at mRNA level. In contrast, protein degradation by IL-1 is an unlikely mechanism to control LRAT expression, or may not contribute to regulation of LRAT expression at substantial level. The key evidence for this notion comes from the fact that ectopically expressed LRAT was devoid of down-regulation by IL-1, suggesting IL-1 does not control LRAT protein degradation. Future work will address how IL-1 suppresses the transcription of LRAT gene. IL-1 can drive four kinase pathways, including IKK, p38 MAPK, JNK, and ERK. Two major transcription factors, NF-κB and AP1, after activation, are recruited to the cis-elements in the targeting genes. The response elements of IL-1 signaling are not apparently found in the proximal region of LRAT, but are evident in distal region of LRAT gene (data not shown). It is openly possible that suppression of LRAT gene is initiated by IL-1-regulated transcription factors that target the cis-elements, which subsequently recruit suppressors/co-suppressors into the gene. Alternatively, decreased mRNA of LRAT may be regulated at mRNA stability. Such scenario is elaborated by the evidence that LRAT mRNA contains 4000 bp 3′-untranslated region (UTR), which is conserved among different species.

Hepatic toxin induced liver damage, in a similar way to other organs, is initiated by activation pattern recognition receptors (PRR) including toll-like-receptors (TLRs), mannose receptors, and NOD-like receptors, which consequently release proinflammatory cytokines such as IL-1 and TNF-α. For instance, a report showed that acetaminophen induced liver injury is mediated by activation of TLR-9 and inflammasome that release mature IL-1 through caspase-1 [29]. Our results suggested that KCs may mediate retinoids storage reduction through secretion of IL-1 (Fig. 6). In addition to KCs, sinusoidal endothelial cells may also provide IL-1 through diverse routes including TLR-9 [29], [30]. Moreover, our preliminary work showed that HSCs in 3D ECM can substantially generate IL-1 in an autocrine manner to amplify the injury signal. Thus, it is possible that the signals triggering mobilization of retinyl esters in HSCs and activation of HSCs may come from diverse sinusoidal cells. Although our attention has been paid to IL-1, TNF-α at a less extent can drive down LRAT expression. Such differential responsiveness to these two cytokines may be derived from the receptor abundance and intracellular signal machinery in a particular cell type. According to our preliminary data, we noticed that, in terms of MMP expression, rat HSCs are more sensitive to IL-1 than TNF-α, whereas mouse HSCs oppositely show higher sensitivity to IL-1 than TNF-α than IL-1.

## Materials and Methods

### Primary hepatic stellate cells and Kupffer cells

HSCs and KCs were isolated from normal male Wistar rats (Charles River, Charleston, CA) using collagenase/pronase digestion and arabinogalactan gradient ultracentrifugation as described previously and provided by the Non- Parenchymal Liver Cell Core of the USC Research Center for Alcoholic Liver and Pancreatic Diseases [31]. The resulting primary HSCs have considerably high purity in 90%, as judged by auto-fluorescence of retinol droplets, desmin and LRAT staining, and star-like morphology. The purity of KCs, also around 90%, is determined by FACS with anti-F4/80 staining. After recovery for 2–3 days following isolation, HSCs were seeded on plastic or embedded in 3D ECM as previously described [13]. All cytokines and growth factors were purchased from R&D systems (Minneapolis, MN).

### Animal model of acute liver injury

B6.129S wild type (WT) mice, and IL-1 receptor deficient (IL-1R^−/−^) mice (B6.129S7-*Il1r1tm1Imx*) (Jackson Laboratory, Bar Harbor, ME) received a single intraperitoneal injection of thioacetamide (TAA; Sigma-Aldrich, St. Lois, MO) at the dosage of 200 µg/g body weight or normal saline as control. Mice were sacrificed at the time points indicated in figures. In one experiment, WT mice were intravenously injected 20 ng of IL-1α or normal saline. To test injury-induced regulation of LRAT in rat model, male Wistar rats (Charles River) were given a single dose of CCl_4_ (Sigma-Aldrich, 1.5 ml/kg body weight, by subcutaneous injection) or mineral oil after priming with 0.5% phenobarbital (Sigma) in drinking water for a week. After 24 hours, liver tissues were harvested for analyses, and HSCs were subsequently isolated from the rat liver tissues [31].

### Ectopic expression of LRAT by adenovirus in rat HSCs

We cloned rat *LRAT* cDNA by reverse transcription of RNA extracts from primary rat HSCs, using following set of primers, 5′-CGGGATCCGCGAGAAACTCTGGTCTTTAAAGGATG-3′ (BamHI recognition site is underlined), and 5′-GGAATTCGGAAGCTAGCCAGACATCATCC-3′ (EcoRI recognition site is underlined). After double-digestion with BamHI and EcoRI, the PCR product was cloned into pAd/CMV/VS-DEST adenoviral vector (Ad.LRAT; Invitrogen, Carlsbad, CA), followed by sequencing confirmation. GFP expressing adenoviral vector was used as control (Ad.GFP). Adenoviral vectors were amplified using 293A cells and purified with cesium chloride gradient centrifugation. On day 2 after initial isolation, HSCs were transduced by Ad.GFP or Ad.LRAT at MOI of 100. On day 5, the cultures were stimulated with or without IL-1α at 10 ng/ml for additional 3 days. For lipid staining, cells were treated with 5 µM retinol (Sigma-Aldrich) and 100 µM palmitate (Sigma-Aldrich) for 24 hours and incubated with 0.2% Oil red O (Sigma-Aldrich) for 5 hours.

### Retinyl palmitate measurement

The procedure for lipid extraction and retinyl palmitate measurement were adopted from others with some modifications [32]. Briefly, liver samples (100 mg) or HSCs (1–2×10^6^ cells) were extracted in chloroform with 0.1% formic acid in glass tubes protected from light. All-trans-retinoic acid was added in extractions as internal standard to trace the systemic variation. After drying up solvent under N_2_, samples were reconstituted in chloroform with 0.1% formic acid, followed by HPLC (Agilent 1100) analysis. Retinyl palmitate and internal standards were detected by Applied Biosystems API 3000 LC/MS/MS triple quadrupole mass spectrometer with APCI source.

### Quantitative RT-PCR (qRT-PCR)

Total RNAs were extracted from rat HSCs or livers using TRIzol reagent (Invitrogen). Two micrograms of RNAs were transcribed using M-MLV reverse transcriptase (Invitrogen) and amplified with qPCR Mastermix Plus for SYBR Green I (Eurogentec, San Diego, CA) as previously described [33]. The primers were listed in Table.

**Table 1:** Primer sequences used for qRT-PCR.

Mouse and rat LRAT:
Forward 5′-ACA AGG AAC GCA CTC AGA AGG T-3′
Reverse 5′-ATG CTG GCC ACC TTG CAA AT-3′
Rat CRBP-I:
Forward 5′-AGG GTG ATG AAC TTC ACC TGG A-3′
Reverse 5′-TTC GGG CTG CTC AGT GTA CTT T -3′
Mouse IL-1α:
Forward 5′-CAC AAC TGT TCG TGA GCG CT-3′
Reverse 5′-TTG GTG TTT CTG GCA ACT CCT-3′
Mouse IL-1β:
Forward 5′-ACT CCT TAG TCC TCG GCC A-3′
Reverse 5′-TGG TTT CTT GTG ACC CTG AGC-3′
Mouse and rat GAPDH:
Forward 5′-GCA CAG TCA AGG CCG AGA AT-3′
Reverse 5′-GCC TTC TCC ATG GTG GTG AA -3′

### DNA Microarray Analysis

Rat HSCs were cultured on plastic or embedded in Col-I and Matrigel respectively [13]. After 24 hour recovery in DMEM with 10% fetal bovine serum (FBS), the cells were challenged by IL-1α at 10 ng/ml in 1% FBS for 18 hours. Total RNA was extracted with Trizol (Invitrogen) and purified by RNeasy Clean up column (QIAGEN, Valencia, CA). RNA was amplified, labeled, and hybridized to the Rat Affymetrix GeneChip Gene 230 Array according to standard Affymetrix protocols.

### Western blot analysis

The procedure for protein preparation from HSCs was previously described [13]. For liver tissue, 100 mg of sample was homogenized in 1 ml of NT buffer (100 mM NaCl, 50 mM Tris-HCl, pH 7.5) by Dounce homogenizer. The homogenate was mixed and boiled with 2× sample buffer for SDS-PAGE with reducing agent. Anti-LRAT mouse monoclonal antibody (at 1∶100), as a gift kindly provided by Dr. Krzysztof Palczewski [19], anti-IL-1β antibody (1∶1000) (R&D systems), anti-α-smooth muscle actin antibody (1∶1000) (α-SMA; Sigma) and anti-glyceraldehydes-3-phosphate dehydrogenase (GAPDH) antibody (1∶1000) (Chemicon, Temecula, CA), and HRP-conjugated secondary antibody (Santa Cruz Biotech, Santa Cruz, CA) were used for Western blot analysis. Blots were visualized by the enhanced chemiluminescence detection system (Thermo, Rockford, IL).

### Immunofluorescence staining

HSCs were fixed by cold methanol followed by permeabilization with 0.1% Triton X100 in PBS and blocked with 10% bovine serum albumin (BSA). Then the cells were incubated with anti-LRAT antibody (1∶100) overnight. To detect primary antibody, the cells were incubated with Cy3 conjugated anti-mouse IgG (1∶500) (Sigma) for 1 hour. Nuclei were stained with 4′,6-diamino-2-phenylindole (DAPI, 1 µg/ml) for 10 min. Cryosections of rat liver were fixed with 4% paraformaldehyde and blocked with 10% BSA in PBS, followed by incubation with anti-LRAT antibody (1∶100) or anti-desmin antibody (1∶200) (Abcam, Cambridge, MA) and fluorophore conjugated secondary antibodies.

### Kupffer cell conditioned medium and treatment of HSCs

KCs seeded on 10-cm dishes were cultured in DMEM with 1% FBS for 3 days to prepare the conditioned medium (KC-CM). In some experiments KCs were additionally activated by lipopolysaccharide (LPS, 5 µg/ml). Then, primary rat HSCs were exposed to KC-CM in presence with or without IL-1 receptor antagonist (IL-1RA) (R&D systems, 1 µg/ml) for 18 hours.

### Gelatinolytic zymography

Liver tissues (100 mg) from the mice subjected to IL-1 challenge or control treatment were extracted in 1 ml of NT buffer (100 mM NaCl, 50 mM Tris-HCl, pH 7.5). The gelatinases in tissue extracts were enriched by gelatin-conjugated Sepharose-4B, followed by zymography [34].

### Statistical analysis

All numerical data were expressed as mean ± S.D. The significance of the differences between two groups was assessed using Student's *t* test. A *P* value of less than 0.05 was considered statistically significant.
